# Capturing the human impact of living with multiple long-term conditions in routine electronic health records – lost in translation?

**DOI:** 10.1177/26335565251329869

**Published:** 2025-04-01

**Authors:** Simon D. S. Fraser, Emilia Holland, Lynn Laidlaw, Nick A. Francis, Sara Macdonald, Frances S. Mair, Nisreen A. Alwan, Michael Boniface, Rebecca B. Hoyle, Nic Fair, Jakub J. Dylag, Mozhdeh Shiranirad, Roberta Chiovoloni, Sebastian Stannard, Robin Poole, Ashley Akbari, Mark Ashworth, Alex Dregan

**Affiliations:** 1 School of Primary Care, Population Sciences and Medical Education, Faculty of Medicine, 7423 University of Southampton, Southampton, UK; 2 University Hospital Southampton NHS Foundation Trust, Southampton, UK; 3 NIHR Applied Research Collaboration Wessex, Southampton, UK; 4 Patient and Public Involvement (PPI) member, MELD-B Project, Southampton, UK; 5 General Practice and Primary Care, School of Health and Wellbeing, 3526 University of Glasgow, Glasgow, UK; 6 School of Electronics and Computer Science, 7423 University of Southampton, Southampton, UK; 7 School of Mathematical Sciences, 7423 University of Southampton, Southampton, UK; 8 Population Data Science, Swansea University Medical School, Faculty of Medicine, Health and Life Science, 7759 Swansea University, Swansea, UK; 9 Southampton City Council, Southampton, UK; 10 School of Life Course and Population Sciences, 4616 King’s College London, London, UK; 11 Institute of Psychiatry, Psychology and Neuroscience (IoPPN), 4616 King’s College London, London, UK

**Keywords:** multimorbidity, long-term conditions, electronic health records, clinical coding, lived experience

## Abstract

**Background:**

Living with multiple long-term conditions (MLTCs) involves ‘work’. A recent qualitative synthesis identified eight patient-centred work themes: ‘learning and adapting’, ‘accumulation and complexity’, ‘investigation and monitoring’, ‘health service and administration’ and ‘symptom’, ‘emotional’, ‘medication’ and ‘financial’ work. These themes may be underrepresented in electronic health records (EHRs). This study aimed to evaluate the representation of these themes and their constituent concepts in EHR data in a general population and among individuals with history of a mental health condition.

**Methods:**

Using the OpenCodelists builder from OpenSAFELY, clinical code lists corresponding to work concepts were developed using Systematised Nomenclature of Medicine Clinical Terms (SNOMED CT) and validated by two clinicians. Additional concepts were engineered within the Clinical Practice Research Datalink (CPRD) and the Secure Anonymised Information Linkage (SAIL) Databank. We analysed trends in recording rates over 20 years across a SAIL general population cohort (n=5,180,602) and a CPRD cohort comprising individuals with a mental health diagnosis (n=3,616,776) and matched controls (n=4,457,225).

**Results:**

55 code lists and seven engineered concepts were developed across the themes. The proportion of patients with codes related to ‘investigation and monitoring’ exceeded 40%, while ‘accumulation and complexity’ and ‘financial work’ were poorly represented (<2% and <1% of the study population respectively). Recording was generally higher among individuals with a mental health diagnosis history.

**Conclusion:**

While EHR data captures some aspects of MLTC work, patient-centred concepts are under-represented. Future research should explore reasons behind variability in coding practices, and innovative methods for enriching structured records with patient-centred data.

## Background

Many studies use anonymised electronic health record (EHR) data sources to investigate patterns and trends in the epidemiology of multiple long-term conditions (MLTCs, often called ‘multimorbidity’).^1–3^ While most commonly-employed definitions of multimorbidity are based on the number of long-term conditions (usually two or more), a 2016 systematic review found that symptoms featured as part of the definition in 71 (62%) of 115 articles reviewed (albeit with lack of consensus about whether certain concepts such as back pain should be considered symptoms or conditions).^4,5^ Similarly, National Institute for Health and Care Excellence (NICE) multimorbidity guidelines recommend that the definition can include ‘symptom complexes such as frailty or chronic pain’ and ‘sensory impairment such as sight or hearing loss’.^6^ NICE also recommends taking a patient-centred, holistic approach to care, including advice to ‘establish disease and treatment burden by talking to people about how their health problems affect their day-to-day life’.^6^ In their consensus study of conditions to include in MLTC research, Ho et al. considered ‘criteria for selecting conditions relating to impact’ and reached agreement on many attributes, such as conditions that reduce quality of life, increase risk of death, worsen self-perceived health status and increase treatment burden.^7^

Despite these analyses and recommendations that go beyond counting conditions, a limitation of EHR studies to date is the under-development of attributes that allow clear consideration of the breadth of experience of living with MLTCs from the patient perspective, including (but not limited to) concepts such as ‘symptom burden’, ‘treatment burden’ and ‘self-perceived health status’. A variety of cluster analyses have shown important distributions that can guide clinical care and health care commissioning priorities, and while methods such as natural language processing/large language models promise to analyse textual medical notes, which often includes patient context, within large numbers of health records, access to clinician-entered free text within confidential medical records is understandably highly restricted and analyses of large datasets are commonly limited to structured record fields.^3,8–13^ This limits the ability to infer a patient-centred/holistic understanding of MLTCs and their impact from clustering studies.

The Multidisciplinary Ecosystem to study Lifecourse Determinants and Prevention of Early-onset Burdensome Multimorbidity (MELD-B) study aimed to develop a deeper understanding of what ‘burdensomeness’ means to people living with MLTCs in order to inform more patient-centred MLTC clustering analyses of anonymised data.^14^ A qualitative evidence synthesis (QES) was conducted that reviewed a substantial body of literature to identify and describe the impact of living with MLTCs on everyday life.^15^ Patient and Public contributors advised that the terms ‘work’ and ‘workload’ were preferred to ‘burden’ and the QES identified eight ‘themes of work’ incorporating multiple concepts characterising the lived experience of MLTCs.^15^ These themes included ‘learning and adapting’ (learning about new and existing conditions and their management, including the physical and psychological adjustments required), ‘accumulation and complexity’ (the additional and cumulative burden of living with multiple, rather than just one, long-term condition), ‘investigation and monitoring’ (the work of tests related to MLTCs), ‘health service and administration’ (work related to navigating health services) ‘medication work’ (work associated with taking and managing medications), ‘financial work’ (the financial impact of living with MLTCs), ‘symptom work’ and ‘emotional work’.^15^ These themes built on and enhanced recognised models such as the Corbin and Strauss ‘three lines of work’, treatment burden and symptom burden.^5,16–19^ The evidence synthesis also highlighted the adverse impact of mental health problems across all themes, which adds to the complexity of living with MLTCs.

This study aimed to develop methods to identify a set of concepts from clinical code lists and engineered variables within EHRs that could represent these themes of work for epidemiological and cluster analyses and potentially for clinical settings. The study also aimed to explore the extent to which work themes and concepts are represented in EHRs in both the general population and within a specific population of people with a history of a mental health diagnosis.

## Methods

The methods of the MELD-B QES have been described elsewhere but, in summary, we searched five bibliographic databases from 2000-January 2023 and included studies where at least 50% of study participants were living with three or more long-term conditions and the lived experience of MLTCs was expressed from the patient perspective.^15^ Quality assessment of studies was undertaken, and data were synthesised using an inductive approach with patient and public involvement colleagues providing input throughout. The eight themes of work that were developed incorporated a large number of individual concepts that were derived directly from the line-by-line coding (in NViVo) of the 46 included qualitative studies involving over 5600 participants.^15,20^ Given the many hundreds of individual concepts identified, it was not practical to develop clinical code lists for all, and it was necessary to take a pragmatic approach to derive a manageable number for this exploratory work.

### Datasets

This study used two datasets within which to explore the recording of these themes and concepts in primary care records, one from the Clinical Practice Research Datalink (CPRD) and one from the Secure Anonymised Information Linkage (SAIL) Databank, the national trusted research environment (TRE) for Wales. In CPRD, a matched prospective cohort was used that had been created for initial use in a related study, which is exploring mental and cognitive disorders-related multimorbidity in linked EHRs. This study involved creating a prospective cohort (which was repurposed for the MELD-B study) that included individuals identified as having an incident mental health diagnosis at any point between 2003 and 2023 (depression, anxiety, dementia, Serious Mental Illness (SMI, schizophrenia, bipolar disorder and psychosis)), matched 1:1 with a randomly selected control group, on age (within a 2-year age band), sex, general practice and index date of mental health diagnosis (for SMI the ratio of cases to controls was 1:2). The controls were allocated the index date for their corresponding case, to ensure matching on calendar time as well. The nature of the CPRD dataset provided an opportunity for comparison in recording of concepts and themes between specific populations, particularly those with a history of mental health disorders, possibly at higher risk of burdensome MLTCs. This dataset was created within the CPRD Portal in the King’s College London ‘CREATE’ Trusted Research Environment.^21^ Within SAIL two cohorts were created: the SAIL MELD-B e-cohort (5,180,602 people between 1^st^ January 2000 and 31^st^ December 2022) and the SAIL MELD-B children and young adult e-cohort (a subset of the SAIL MELD-B e-cohort including only individuals born on or after the cohort start date).^22^ The SAIL MELD-B e-cohort was used for these analyses.

### Concept development

The concepts were discussed within the MELD-B team, and then one investigator (SF) with extensive experience of clinical coding in primary care as a General Practitioner (GP) searched the clinical terms lists that related to concepts from the QES in the ‘OpenCodelists’ builder tool.^23^ OpenCodelists is a facility that supports the creation and sharing of clinical code lists and allows searching under four code types: Systematised Nomenclature of Medicine Clinical Terms (SNOMED CT), International Classification of Diseases 10th Revision (ICD-10), Read Clinical Terms version 3 (CTV-3) and Pseudo British National Formulary (BNF).^23–25^ We used SNOMED CT, which is a widely adopted, structured clinical vocabulary for use in EHRs, used in over eighty countries including the National Health Service in England.^24,26^

Clinical code lists representing work concepts were generated, and the emerging lists were reviewed with a second member of the team (AD) before being sent to a second clinical reviewer (EH, NF, MA) within the team who undertook a verification process of each code, considering whether the code list correctly reflected the concept being captured and whether other potential codes were known that should be added. If any additional codes were suggested, the OpenCodelists were searched again, and any relevant new codes were included. In this way, all codes in all code lists were reviewed by at least two clinicians with experience in clinical coding in primary care.

**Figure 1.**
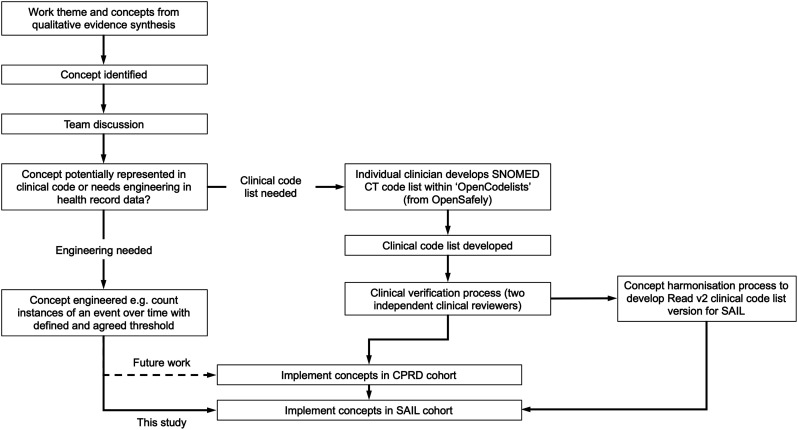
Overview of the process for deriving concepts relating to themes of work in data.

A number the concepts were not represented by clinical code lists but needed to be engineered within EHRs. For example, ‘numbers of GP appointments’ within the ‘health service and administration’ theme of work, would not be reflected by a clinical code list but can be calculated by counts within the relevant field within EHRs. A similar approach was implemented for characterising numbers of medications within the ‘medications’ theme. The complete code lists for each concept are available via https://git.soton.ac.uk/meldb. Seven of these engineered concepts were used as exemplars for these descriptive analyses and applied only in the SAIL cohort in this exploratory study. In the SAIL Databank, primary care data are coded using Read version 2.^27^ As Read v2 is now retired for most of the UK, there was therefore an extra step required to map code lists identified in SNOMED CT to Read v2. The code lists were uploaded to a shared repository and then processed using an automated concept harmonisation pipeline. The pipeline provided the clinical team with an efficient and reproducible process for the publication of versioned mappings from source to target code lists, including deltas between mapping versions as concepts and code lists are iterated and revised. The pipeline normalised source code lists into a common format before verifying codes against NHS TRUD registered codes.^28^ The NHS TRUD data migration mappings were then used to translate SNOMED CT to Read v2, including missingness reports. The concept code lists were then published and versioned in a git repository for SNOMED CT to Read v2. The concepts were then incorporated into the SAIL MELD-B e-cohort (SMC) to select patients and create research-ready data tables. These were labelled with version control so team members creating and using the tables were clear about the version of concepts with which they were working. An overview of the process is shown in Figure 1.

Having imported the concepts into each of the cohorts, their distributions were explored using counts and proportions per cohort year, including descriptive comparisons between ‘cases’ (those with a history of a mental health diagnosis) and ‘controls’ (those without) in the CPRD cohort. The proportion of the population with any record of each concept and theme was plotted per mid-year population of the cohorts for each year. Mid-year population was constructed as an annual figure based on the total population of CPRD contributing data on the 30th of June each study year. A theme (e.g. ‘Emotions’) was considered present during a specific study year, if a relevant code for a concept (e.g. ‘low self-esteem’) indicative of the theme was recorded in a patient EHR during that year.

**Figure 2.**
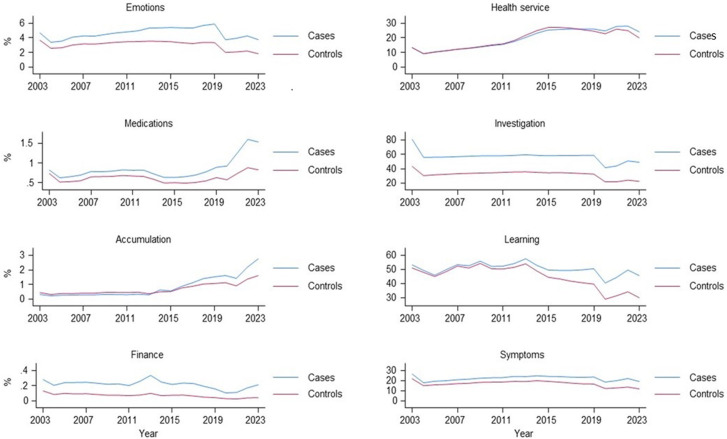
Trends in recording of the eight work themes within the CPRD cohort. Footnote: Numerator - any occurrence of the concept in a year, denominator: mid-year cohort population, Accumulation = ‘Accumulation and complexity’ theme, Learning = ‘Learning and adapting’ theme, Investigation = ‘Investigation and monitoring’ theme, Health service = ‘Health service and administration’ theme, Cases = those an incident mental health diagnosis at any point between 2003 and 2023. Controls = randomly selected group matched 1:1 with controls.

For ‘Medications’, a single prescription was not considered sufficient for an individual to be included in the medication proportions. To reflect chronicity of medication use, an individual needed to have at least one prescription for a specific medication in at least three out of four quarters of a year. Once this threshold was met, the number of such medications for each individual within that year was counted. If an individual had at least one such medication, they were included in the cohort population reported in the “medication” figure (Figure 2).

For this exploratory study, we did not rely on records explicitly Read-coded as “Pain” in the data. The engineered concept “Pain” was identified through the prescription of pain medication, specifically having more than three prescriptions of any pain medication per year.

For did not attend (‘DNA’), accident and emergency attendance (‘A&E’), interaction with a GP (‘GP’), hospital admissions, and outpatient appointments, we counted the number of individuals with at least one recorded event in the database for each specific case within a given year.

In SAIL, data for accident and emergency (also known as emergency department data) and outpatient appointments only began in 2009 and 2004 respectively, meaning that there were no records in the cohort prior to those years, resulting in zeros in the plots. Additionally, in SAIL, there was no way to isolate records specifically related to face-to-face GP consultations, so all GP interactions, including administrative tasks, consultations (of any kind), investigations, prescriptions, and referrals were considered together.

## Results

From the eight themes of work, 55 code lists were developed, reflecting aspects of each theme. A further eleven concepts were engineered within the available data, of which seven were included in these analyses. A summary of the concepts (from code lists and engineered in data) are shown in Table 1. The concepts cover all eight work themes, with variation in the extent to which code lists (and therefore concepts) were available within the themes.

**Table 1.** Summary of clinical code lists and engineered concepts for each theme.

**MELD-B qualitative evidence synthesis themes of work**	**Concepts represented by clinical code lists in CPRD data**	**Engineered concepts in SAIL** (only those in **bold** were included in these descriptive analyses)
**Accumulation and complexity**	Care coordination Drug interaction Self-advocacy/self help	Rate of accrual of long-term conditions Total number of long-term conditions
**Emotional work**	Anger Despair Embarrassment Fear Frustration Guilt Low self esteem Sadness Shame Stress Suicidal thoughts	
**Financial work**	Benefits Income	Socioeconomic status (from index of multiple deprivation)
**Health service and administration**	Admission to hospital Did not attend (DNA)	**Number/frequency of interactions with primary care** **Number/frequency of outpatient appointments** **Number/frequency of accident and emergency (A&E) attendances** **Number/frequency of hospital admissions** **Number/frequency of ‘did not attends’ (DNAs)**
**Investigation and monitoring**	Blood pressure reading Drug monitoring Long-term condition monitoring Physical examination Respiratory tests	Number of blood tests
**Learning and adapting**	Alcohol advice Bereavement Diet advice Employment problem Housing problem Lifestyle advice Needs help Not coping Physical activity advice Smoking status assessed Weight loss advice	
**Medication work**	Adherence problems Difficulty with medication Drug adverse effect	**Number of medications**
**Symptom work**	Breathlessness Chest pain Confused Cough Falls Gait problems Gastrointestinal pain Headache Memory problems Mobility problems Nausea Reduced appetite Reduced physical strength Sleep problems Sweating Tiredness Unintentional weight loss Urinary incontinence	**Pain (derived from prescriptions)**

### CPRD findings

Table 2 gives a summary of the demographic characteristics of the CPRD cohort. Those with a history of mental health diagnosis (‘cases’, n=3,616,776) and their controls (n=4,457,225) were closely matched with regards to age, sex, practice, and region of residence (government office region). The majority of cases and controls were below age 40 at the time of mental health condition diagnosis, with a higher proportion of women than men (61% vs 39%), and were more likely to reside within North-East (22%), South-East (22%), or West Midlands (17%) than other regions. When considering specific mental health conditions, there were notable differences, particularly with regards to age at diagnosis. For example most patients were over 70 years of age at the time of dementia diagnosis. Among people with a SMI diagnosis, London, the South East and the North West were more strongly represented than other regions.

**Table 2.** Demographic characteristics of the CPRD cohort, both overall (cases and controls) and mental health condition-specific.

		Overall		Mental health condition history among cases^b^			
		Controls n (%)	Cases n (%)^a^	Depression n (%)	Anxiety n (%)	Dementia n (%)	Serious mental illness n (%)
	Total	4,457,225	3,616,776	1,967,422	2,065,130	442,948	128,807
Age groups	<30	1,388,422 (33)	1,066,793 (30)	617,056 (31)	733,515 (36)	42 (0)	35,624 (27)
	30-39	837,641(20)	635,222 (18)	400,782 (20)	400,953 (19)	102 (0)	25,800 (20)
	40-49	712,553 (17)	547,536 (15)	346,364 (18)	336,089 (16)	706 (0)	22,030 (17)
	50-59	538.451 (13)	419,485 (12)	260,867 (13)	255,036 (12)	5,213 (1)	16,030 (13)
	60-69	313,543 (7)	267,615 (7)	144,617 (7)	154,768 (7)	23,645 (5)	10,750 (8)
	≥70	412,636 (10)	680,125 (19)	197,922 (10)	184,499 (8)	413,241(94)	20,305 (17)
Age	Mean(sd)	41 (19)	46 (22)	42 (18)	40 (18)	82 (8)	45 (20)
Sex	Female	2,534,907 (60)	2,209,056 (61)	1,169,456 (60)	1,313,949 (63)	272,602 (52)	66,409 (52)
	Male	1,668,259 (40)	1,407,506 (39)	797,835 (40)	751,026 (37)	170,344 (48)	62,398 (48)
Region	North East	157,323 (4)	150,759 (4)	77,185 (4)	89,787 (4)	18,358 (4)	4,758 (4)
	North West	860,745 (22)	795,929 (22)	436,527 (22)	480,566 (23)	94,856 (22)	28,023 (22)
	Yorkshire	130,146 (3)	123,285 (3)	64,310 (3)	72,279 (4)	14,659 (3)	3,374 (3)
	East Midlands	95,522 (2)	88,559 (2)	48,266 (2)	51,673 (3)	9,953 (2)	2,417 (2)
	West Midlands	649,042 (17)	608,829 (17)	339,815 (17)	346,403 (17)	74,323 (17)	19,803 (15)
	East Anglia	136,831 (4)	132,487 (4)	68,008 (3)	74,878 (4)	17,707 (4)	4,053 (3)
	London	563,514 (15)	525,736 (15)	285,807 (15)	294,319 (14)	55,326 (13)	28,439 (22)
	South East	833,793 (22)	789,486 (22)	439,529 (22)	431,430 (21)	100,954 (23)	25,711 (20)
	South West	397,655 (11)	383,951 (11)	200,413 (10)	215,182 (10)	52,255 (12)	10,800 (8)
	Northern Ireland	15,089 (0)	13,578 (0)	7,562 (0)	8,613 (0)	1,288 (0)	432 (0)

^a^numbers are frequencies (n) and column percentage unless stated otherwise.

^b^rows do not add up to the cases total because individuals may have had more than one condition in the past.

Figure 2 shows the distribution and trends in recording the eight themes during the study period in the CPRD cohort.

The findings revealed some noteworthy patterns of use over time and across themes. A non-linear distribution of all themes’ use was observed, with a steady increase in recording rates to around pre-COVID-19 pandemic time (or before), followed by a slight decline in the early years of the COVID-19 pandemic, and then a modest increase towards the end of the follow-up period. For instance, Investigations and Monitoring theme concept recording increased from around 56% in 2003 to around 59-60% in 2019 among those with a mental health diagnosis, followed by a sharp drop to 42% in 2020, and then a gradual increase to 50% by end of 2023. Not all themes followed this trend, however. Health Service and Administration, for example, showed a rather linear increase from 9% in early 2000s to around 28% by 2022, followed by a slight drop to 24% in the most recent year (2023). Symptoms were much more commonly recorded than emotions for both cases and controls, with around a fifth to a quarter of patients with mental health conditions having codes for these themes recorded during the study period. Concepts within the Finance theme showed the lowest rate of recording across all study years.

Supplementary figures show trends in specific concepts within specific themes in CPRD. Similar to the eight themes’ distribution in Figure 2, there was substantial heterogeneity in trends for concept recording over time and across different concepts. For example, there was a steady upward trend in the recording of drug monitoring codes over the study period for the Investigation and Monitoring theme (Supplemental Figure S1(a)), whereas an inverse U-shaped trend was observed for the use of LTC monitoring codes. Of note, while on average those with mental health conditions (cases) showed greater use of codes, this was not uniformly observed. For example, the controls showed consistently greater recording of physical examination codes compared to those with a history of mental health diagnoses. Moreover, the Covid-19 pandemic period coincided with a sharp drop in recording across most concepts, with some exceptions (e.g. Care coordination within the Accumulation & Complexity theme (Supplemental Figure S1(b)) and Did Not Attend (DNA) within the Health Service and Administration theme (Supplemental Figure S1(c)). Codes for Financial work had the lowest rate of use across the study period (Supplemental Figure S1(d)).

### SAIL findings

**Figure 3.**
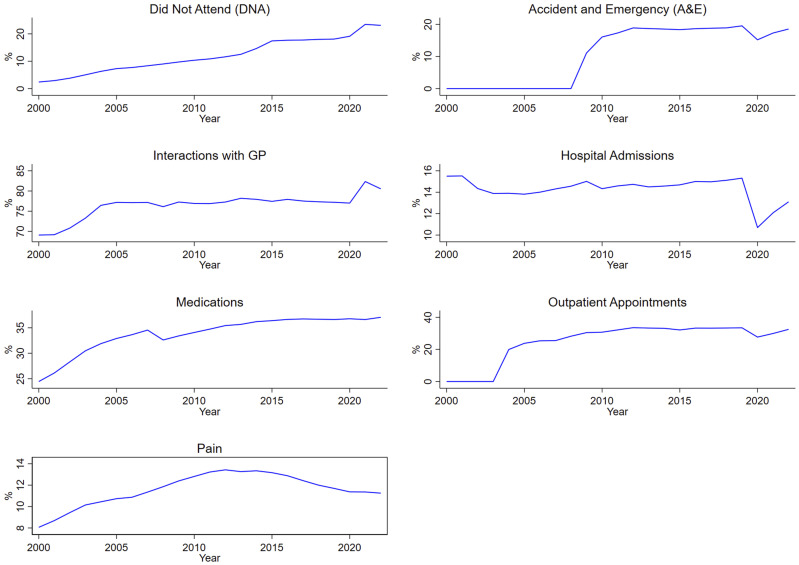
Trends in recording of seven engineered work concepts within the SAIL cohort.

The characteristics of the SAIL MELD-B e-cohort have been described elsewhere, but in brief, this longitudinal cohort used records from linked health and demographic data sources for individuals with available records at any time between 1 January 2000 and 31 December 2022.^22^ It comprises 5,180,602 individuals (50.3% female, 49.7% male) age 0 to 105, and includes 90 LTCs. Figure 3 shows the trends in recording of seven engineered work concepts within this SAIL cohort.

Most of these showed little change in recording over the cohort period, though ‘Did not attend’ showed a gradual increase over time as did ‘Medications’, and ‘Pain’ showed a slight inverse U-shape trend. ‘Hospital admissions’ showed a distinct drop around the time of the pandemic, while ‘Interactions with the GP’ showed an increase.

## Discussion

In two nationally representative cohort studies, we have documented the challenges and opportunities associated with characterising novel multidimensional measures of MLTC work in EHRs. As expected, given the nature and primary purpose of EHRs, patient-focused work concepts (such as emotions, accumulation and complexity) tended to be less well captured, while clinically-driven concepts (such as ‘investigation and monitoring’ and ‘medications’ (based on prescribing)) had a greater level of recording. A key finding emerging from this study was the substantial heterogeneity in recording both between and within the eight work themes over time. Such heterogeneity may mirror the patterning of MLTC trajectories over time, an area we propose to investigate in future work.

In CPRD our findings identified a higher prevalence of specific themes in people with mental health conditions relevant to their matched cohort without mental health diagnoses. This finding emphasises that the burden of MLTC is likely higher among patients with co-existing mental and physical LTCs, and suggests that this group should be prioritised for prevention and intervention. It may be that people with mental health problems consult more often for their health and thus have more opportunities to have symptoms and diagnoses assessed and recorded (though we did not demonstrate a difference in the ‘health service’ theme in these analyses). Mental health conditions are more common in more socioeconomically deprived populations, they may interfere with daily functioning, psychotropic medications have side effects that can contribute to higher symptoms rates, and people with mental health conditions have higher rates of co-occurring physical LTCs.^29^ These factors may exacerbate or contribute to the higher representation of symptoms, medications, emotions, finance, accumulation and investigation themes seen in our analyses.These findings resonate with recent studies looking at the association of different MLTC clusters, such as those involving chronic pain and depression, with large deficits in health-related quality of life.^30^

Strengths of our study include the robust nature of the QES on which the work was based, the broad range of concepts derived, the two-clinician verification process for clinical code lists and the exploratory nature of the descriptive analyses in two large datasets, with diverse clinical and demographic characteristics for greater generalisability.

**Figure 4.**
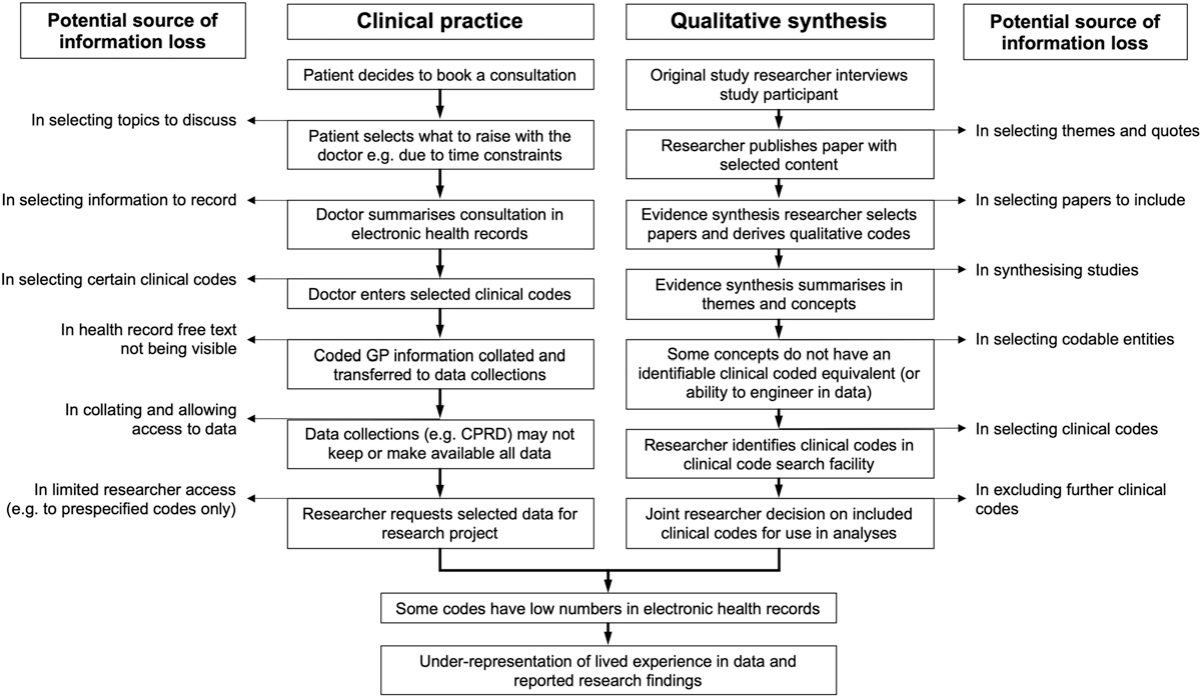
Information loss and risk of bias between clinical consultations, qualitative research and data analysis in electronic health records.

In order for this field to progress, however, it is vitally important to adopt a transparent recognition of the significant limitations. In developing our methods, it was recognised at an early stage that the potential for information loss at many stages could lead to potential bias through selection of specific concepts and exclusion of others. We recognise, for example, the selection bias inherent in not finding clinical code terms related to some of the concepts identified in the QES, in the limited number of codes available for some concepts, in the omission of some concepts from some themes (e.g. inability to include every symptom or every emotion) and so on. Figure 4 summarises the steps in the process from qualitative data collection and from a clinical encounter to expression of concepts in data and highlights some areas of potential information loss.

We also recognise the potential for lack of precision in the definitions of some concepts that arises from the structured nature of coding terminology such as that used in SNOMED CT. On the other hand, SNOMED CT codes do afford a standardised approach to coding clinical information by clinicians that should facilitate comparative international investigations. The variation in expression of these concepts when applied to large routine data cohorts likely therefore reflects both variation in use of such codes and variation in the underlying concept itself, with under-recording of many burden-related codes being likely in clinical practice as suggested by our findings.

Given that clinical consultations are often time-pressured and their primary purpose is to address clinical problems rather than to record experiences for research, our findings are perhaps not surprising. However, patient or person-centred care is recognised as being central to good clinical practice, and patients’ views and experiences of recording practices of data that is about them is unclear.^31^ Moreover, information that is entered into primary care records is constantly evolving, leaving potential for future development of coding practices to better reflect the impact of living with long-term conditions. Many studies have explored, directly or indirectly, aspects of the impact of living with MLTCs in EHRs for more readily-measured attributes such as polypharmacy and health service use.^9,32^ As noted above, other non-UK MLTC studies have incorporated elements of symptoms in their definitions.^5^

We are not aware of studies trying to capture the complexity of MLTCs lived experience using structured EHRs. In addition to the loss of information in the journey from the primary care consultation to data, it is worth considering the likely lack of recording of many aspects of true patient experience in primary care records. Seminal evidence from qualitative studies of doctor patient communication in primary care have described the importance of incorporating ‘the voice of the lifeworld’, in other words ‘the patient’s contextually-grounded experiences of events and problems in their life’ not just ‘the voice of medicine’ in medical encounters.^33,34^ However, from Barry et al’s work on the agenda patients bring to GP consultations, there is evidence that patients often do not voice their real agenda.^34,35^ In these studies symptoms were commonly voiced, while worries about possible diagnoses, what the future holds, side effects, not wanting prescriptions, information on social context were often not.^35,36^ The ‘biographical work’ which Corbin and Strauss describe in terms of ‘the continual or occasional reconstruction of his or her life’ associated with living with MLTCs is very unlikely to be clearly represented in EHRs, but is central to individuals’ experience.^16^

In exploring the impact of MLTCs using concepts derived from qualitative research, we have identified how challenging it is to identify this ‘voice of the lifeworld’ in EHRs. GPs may record some of the relevant information as clinical notes rather than structured EHRs, but such free text is not available to researchers in the UK without individual patient consent. This limits analyses of MLTC clusters and their outcomes and reduces the ability for health services to achieve goal-concordant care (defined as care aligned with a patient’s known goals and values) that takes into account the priorities of patients in addition to clinical needs.^37^ An important finding from Barry’s 2001 study of 35 GP consultation case studies remains highly pertinent: ‘The real problems seem to lie in the consultations where patients were consulting about chronic physical problems. To patients these conditions were a lifeworld issue. However, the doctors seemed to see them as a physical issue requiring the voice of medicine, and the blocking or ignoring of the voice of the lifeworld as a nuisance or an inconvenience’.^34^

A further important aspect to note is that in both clinical practice and research, the term ‘complexity’ is sometimes used interchangeably with ‘multimorbidity’.^38^ There is lack of consensus in what complexity means, including both medical and non-medical aspects, differing numbers of conditions, involvement of different body systems, and when physical, psychological and social issues interact.^38^ The perspective is commonly that of the clinician, not the patient, and there is limited understanding on the personal experience of ‘complexity’. Compounding this further, the MELD-B QES identified significant lack of patient and public involvement and engagement (PPIE) within its included studies and so this study incorporated substantial PPIE in the conceptualisation of the themes and discussion around the concepts and the risk of bias arising from aspects not represented in data. Risks associated with using EHRs have been clearly described and include selection bias, imprecise variable definitions and variable measurement frequency, all accusations that could be levied at this work.^39^ We therefore regard this as a first, exploratory step in the process of recognising, describing and starting to address that lack of true patient-centred representation in EHR data.

Looking to the future, some studies have started to explore the potential of remote symptom monitoring for long-term conditions such as cancer, rheumatoid arthritis and epilepsy and integrating these into EHRs.^40^ There is potential for such remote recordings to be used to enhance clinician understanding of the symptom aspect of MLTCs impact. There is also potential for AI methods to ‘listen’, capture and code work/burden concepts within the clinical consultations, obviating the need for clinicians to manually enter codes.^41^ Natural language processing models have also been used to explore the recording of discussions about finance within primary care consultations.^42^

‘Symptom science’ is a related field historically focusing specifically on symptoms, but with recent suggestions to expand to a broader concept of patient-centred experience as well as addressing policy and population health.^43^ There is ongoing work to link EHRs with administrative data (e.g. employment, education, social care etc.), such as within the SAIL Databank, which should help address the completeness of some of the non-clinical concepts explored in this study.^44^

The MELD-B collaboration is taking a lifecourse approach to MLTC prevention and impact mitigation that will include consideration of clusters centred around concepts that relate to the burden, work and impact of MLTCs, not just clinical outcomes, as well as identifying key early life determinants.^14,45^ As part of this, a consensus process is being undertaken that will identify which concepts are most important to patients and carers in terms of the work they involve and, among clinicians, the likelihood of these concepts being coded in primary care, helping to further validate these concepts for use in MLTC research.^14^ ‘Work’ and ‘burdensomeness’ have an important impact on quality of life, adherence to treatment, and thereby clinical outcomes and improving the ability to capture the lived experience in clinical encounters will facilitate development of relevant interventions. Methodologically, MELD-B also offers novel insights into the biases inherent when combining data from multiple sources and will propose solutions to minimise their impact in MLTC research.

## Supplemental Material

Supplemental Material - Capturing the human impact of living with multiple long-term conditions in routine electronic health records – lost in translation? Supplemental Material for Capturing the human impact of living with multiple long-term conditions in routine electronic health records – lost in translation? by Simon D. S. Fraser, Emilia Holland, Lynn Laidlaw, Nick A. Francis, Sara Macdonald, Frances S. Mair, Nisreen A. Alwan, Michael Boniface, Rebecca B. Hoyle, Nic Fair, Jakub J. Dylag, Mozhdeh Shiranirad, Roberta Chiovoloni, Sebastian Stannard, Robin Poole, Ashley Akbari, Mark Ashworth and Alex Dregan in Journal of Multimorbidity and Comorbidity

## Data Availability

The data used in this study were obtained from two sources - the SAIL Databank at Swansea University, Swansea, UK and the Clinical Practice Research Datalink (CPRD). All proposals to use SAIL data are subject to review by an independent Information Governance Review Panel (IGRP). Before any data can be accessed, approval must be given by the IGRP. The IGRP carefully considers each project to ensure the proper and appropriate use of SAIL data. When approved, access is gained through a privacy-protecting trusted research environment (TRE) and remote access system referred to as the SAIL Gateway. SAIL has established an application process to be followed by anyone who would like to access data via SAIL https://www.saildatabank.com/application-process. CPRD data sources are made available for scientific and medical research after submission of a study protocol to be reviewed and approved by the CPRD Research Data Governance (RDG) Process. Owing to ethical restrictions, the data used in this analysis are not publicly available, in line with the data privacy rules set up by CPRD https://www.cprd.com/privacy-notice. Data access queries can be directed to enquiries@cprd.com.

## ORCID iDs

Simon D. S. Fraser https://orcid.org/0000-0002-4172-4406

Emilia Holland https://orcid.org/0000-0001-5722-3836

Lynn Laidlaw https://orcid.org/0000-0001-6688-6658

Nick A. Francis https://orcid.org/0000-0001-8939-7312

Frances S. Mair https://orcid.org/0000-0001-9780-1135

Michael Boniface https://orcid.org/0000-0002-9281-6095

Rebecca B. Hoyle https://orcid.org/0000-0002-1645-1071

Sebastian Stannard https://orcid.org/0000-0002-6139-1020

Robin Poole https://orcid.org/0000-0002-3113-5202

Ashley Akbari https://orcid.org/0000-0003-0814-0801
