# Supplementary material for: Capturing the human impact of living with multiple long-term conditions in routine electronic health records – lost in translation?
Source: J Multimorb Comorb. 2025 Apr 1;15:26335565251329869. doi: 10.1177/26335565251329869 (PMC11963726; doi:10.1177/26335565251329869)

Supplementary material

Note: In the following figures, cases and controls are defined as:
Cases: Individuals with an incident mental health diagnosis at any point between 2003 and 2023 (depression, anxiety, dementia, Serious Mental Illness (SMI, schizophrenia, bipolar disorder and psychosis))
Controls: a randomly selected control group, matched 1:1 on age (within a 2-year age band), sex, general practice and index date of mental health diagnosis (for SMI the ratio of cases to controls was 1:2)

Supplementary Figure S1a. Trends in recording for individual concepts within the Investigation and Monitoring theme in CPRD

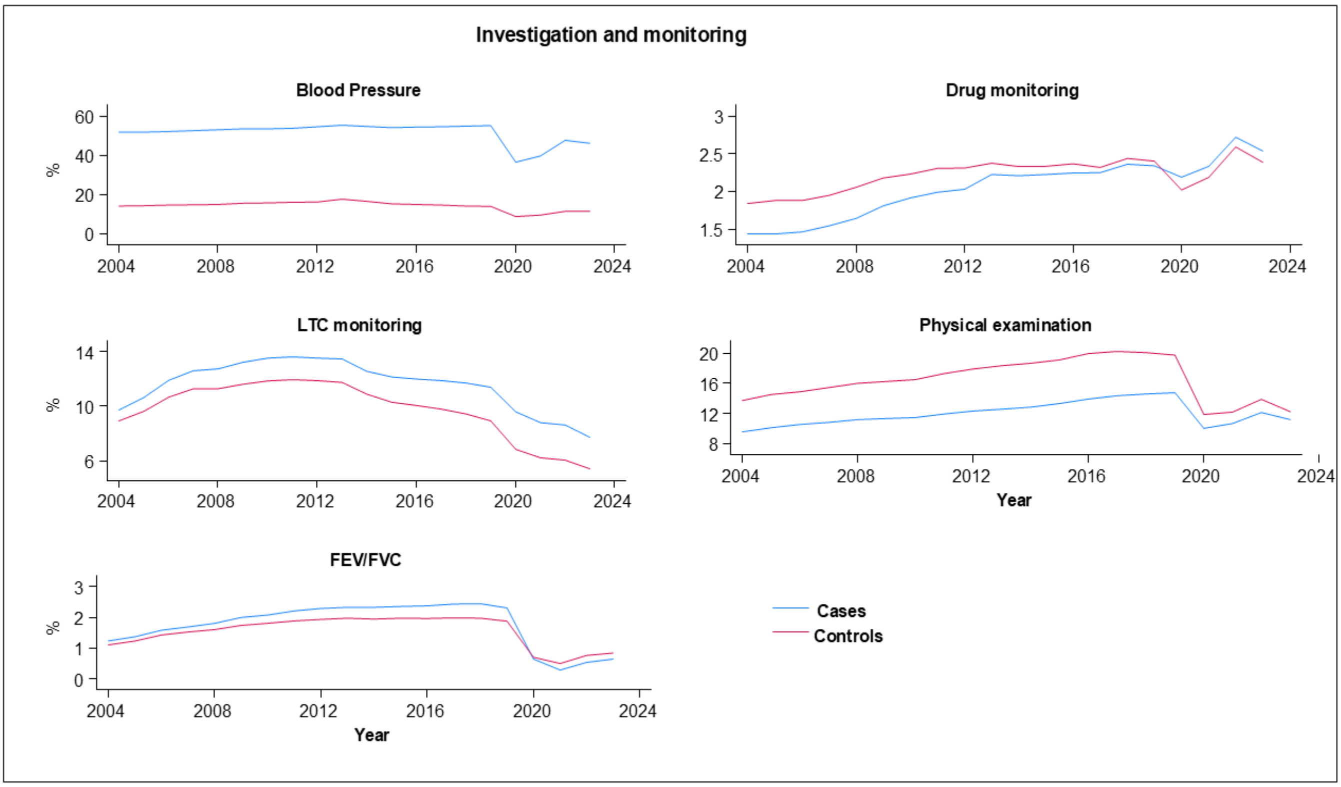

Supplementary Figure S1b. Trends in recording for individual concepts within the Accumulation and Complexity theme in CPRD

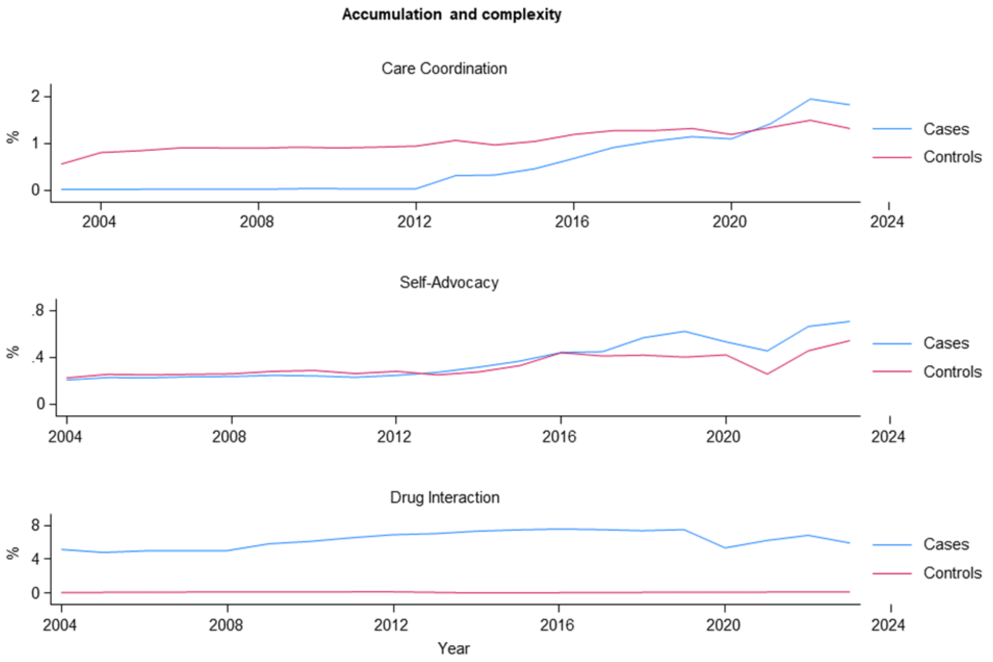

Supplementary Figure S1c. Trends in recording for individual concepts within the Health service and Administration theme in CPRD

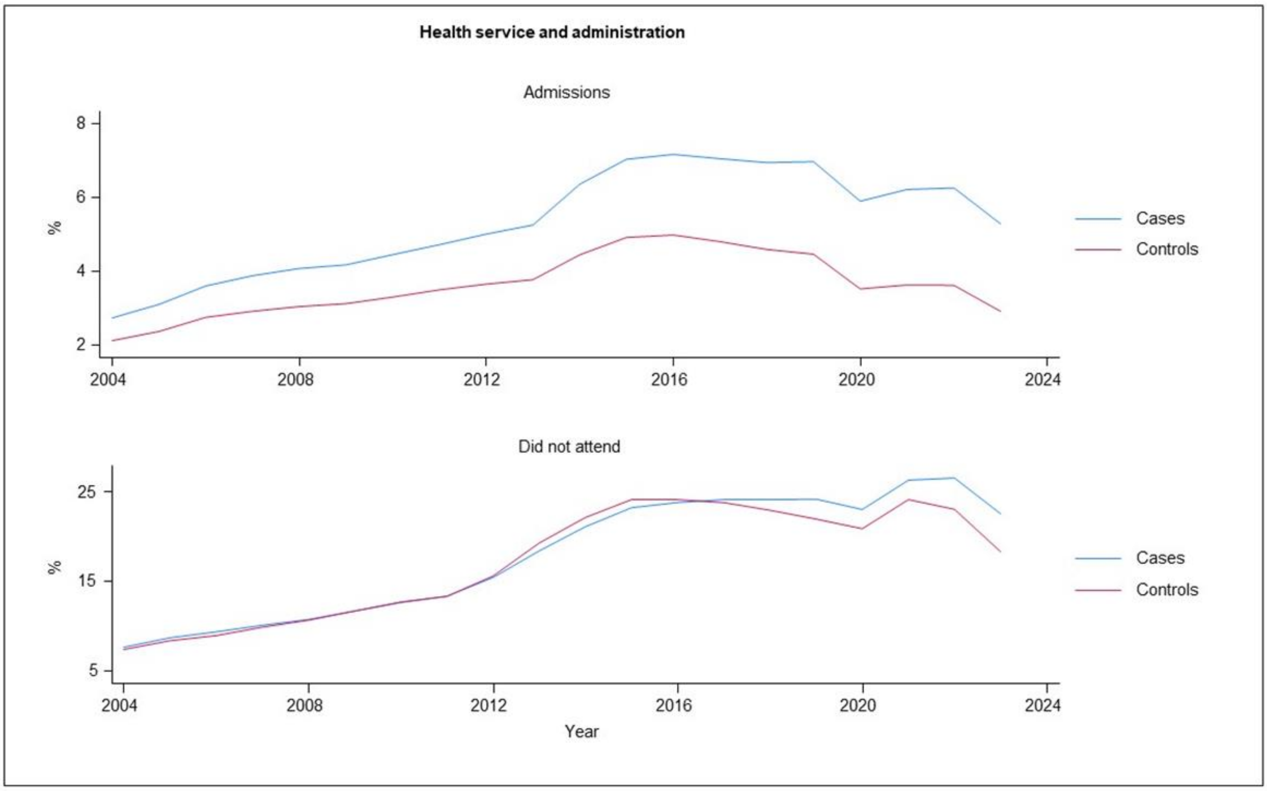

Supplementary Figure S1d. Trends in recording for individual concepts within the Finance theme in CPRD

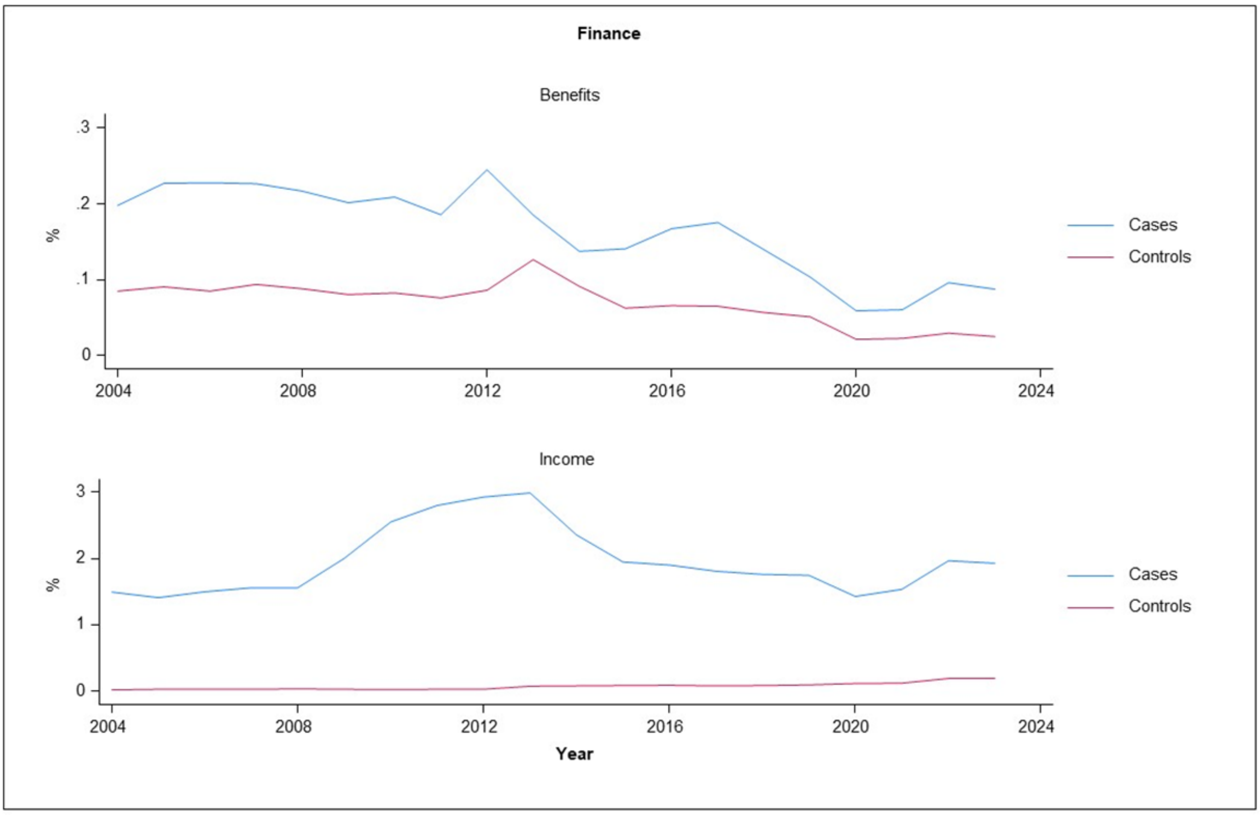

Supplement: Supplemental Material - Capturing the human impact of living with multiple long-term conditions in routine electronic health records – lost in translation? [file sj-pdf-1-cob-10.1177_26335565251329869.pdf]
